# Notes and News

**Published:** 1897-08-07

**Authors:** 


### NOTES AND NEWS.

(Collected and Communicated.)

New wards for the treatment of the diseases of women have been added to the Broca Hospital, in Paris. This supplies a want in the gynæcological teaching of the Paris Medical Faculty. The Broca Hospital, under Dr. Prozzi's direction, acts as a pioneer in several directions relating to hospital management. Special attention has been paid to the decoration of the wards, and their sanitation and ventilation are regarded as excellent.

Paris has been scared from the consumption of rabbits at the present time from a rather amusing cause. Some housebreakers are reported to have stolen 23 rabbits from a bacteriological laboratory in the city, where they were kept for the culture of the germs of cholera, plague, diphtheria, and anthrax. Information was immediately given to the police, with the comforting assurance that if the rabbits are well cooked no infection is likely. We imagine, all the same, the trade in rabbits will be dull in Paris for some time to come.

Patriotism and loyalty have received much stimulation through the Jubilee, and anything which tends towards improving England as a nation is not without interest at the present time. We therefore do not hesitate to point out a field new to many generously-disposed people in the form of an organisation not nearly so well known as it should be. This is the King's Royal Rifle Cadet Corps, a volunteer band of boys between the ages of sixteen and eighteen, recruited from some of the poorest parts of London. The battalion receives no grant from Government; the movement is a pioneer one, and dependent upon the charitable public for its support. The good effected by the discipline, training, and interest afforded has the best results. Every Monday and Friday evening drill takes place in the Guildhall, and in the summer a seaside camp is installed, where a fortnight is spent by the cadets. The expenses are very heavy, and Lieut.-Colonel Wills will thankfully receive contributions at 2, Finsbury Square, E.C.

Leprosy is so prevalent in Panama that a leper refuge is a necessity. At present the attempts at segregation are most elementary, and the lazar home just without the city of Panama is extremely inadequate. The sufferers gathered there have, we understand, neither medicine nor medical attendance, whilst the number at large is unknown. The Government talks of making Coiba Island a refuge.

We are glad to see that the Distribution Committee of the Hospital Sunday Fund state in their report that they have already available for distribution about £40,000, of which £39,706 will be paid to the hospitals and dispensaries as against £41,829 distributed last year. There is, therefore, every reason to hope that the total sum which will be received by the Hospital Sunday Fund in 1897, when all the collections are paid in, will equal, if it does not even exceed, the average of former years. It is satisfactory to find, despite the gloomy forebodings to the contrary, that the Prince of Wales's Fund has not, therefore, interfered with the contributions to the hospitals from the congregational collections made throughout the Metropolis.

It is perhaps a unique occurrence that a medical officer of health should have to announce that a church is unfit for human habitation. Nevertheless this occurrence has actually taken place at the parish church of Hanwell, past which the river Brent flows. According to a warning received from the medical officer of health, the rector had to inform his congregation one Sunday morning that the river was at the time no better than an open sewer, and that its vicinity was dangerous. The very patent fact has caused much alarm in the neighbourhood, and many prominent residents have removed their households already. In the meanwhile the cumbrous official machinery has been set going, which is to determine what steps can be taken to remedy the deplorable condition of the river, and prevent depopulation of the surrounding district by removal or epidemic.

St. Mary's Hospital will be closed during the months of August and September. The floors of several of the wards in the old building, especially the surgical wards, are in a very defective state, and are to be taken up and replaced with teak flooring. The operation theatre is to be reconstructed and refitted, and the whole interior of the hospital is to be redecorated. The out-patient department is to remain open until September 15th, when it will be closed for one month, at the expiration of which time the new out-patient department, forming the basement floor of the Clarence Memorial Wing, will be ready for occupation. Arrangements have been made to send the convalescent patients to convalescent homes, and to provide for the others in neighbouring hospitals. The builders take possession of St. Mary's Hospital on August 4th, and it is expected that the wards will be ready for the reception of patients again on October 1st. The secretary states that the expenses of the works referred to will be heavy, and that the financial difficulties which have surrounded St. Mary's Hospital during the last four years are great. The committee therefore hope that many persons may be found to send liberal contributions to the hospital between now and the end of the present year. We heartily endorse this appeal.

